# From “Local Control” to “Dependency”: Transitions to Single-Vendor Integrated Electronic Health Record Systems and Their Implications for the EHR Workforce

**DOI:** 10.1007/s11606-023-08281-6

**Published:** 2023-10-05

**Authors:** Julian Brunner, Ekaterina Anderson, David C. Mohr, Adena Cohen-Bearak, Seppo T. Rinne

**Affiliations:** 1 Center for the Study of Healthcare Innovation, Implementation & Policy, VA Greater Los Angeles Health Care System, Los Angeles, CA USA; 2 Center for Healthcare Organization and Implementation Research, VA Bedford Healthcare System, Bedford, MA USA; 3 Department of Population and Quantitative Health Sciences, University of Massachusetts Medical School, Worcester, MA USA; 4 Center for Healthcare Organization and Implementation Research, VA Boston Healthcare System, Boston, MA USA; 5 Department of Health Policy and Management, Boston University School of Public Health, Boston, MA USA; 6 Pulmonary & Critical Care Medicine, School of Medicine, Boston University, Boston, MA USA

**Keywords:** electronic health record transition, implementation, workforce, autonomy, veterans, qualitative research

## Abstract

**Background:**

Healthcare systems that previously used either a single legacy electronic health record (EHR) system or a “best-of-breed” combination of products from multiple vendors are increasingly adopting integrated, single-vendor EHR systems. Though healthcare leaders are beginning to recognize the dramatic collateral consequences of these transitions, their impact on the EHR workforce — internal actors most closely involved in governing and supporting the EHR — is poorly understood.

**Objective:**

Identify perceived impacts of adopting single-vendor, integrated EHR systems on the institutional EHR workforce.

**Design:**

In this qualitative study, we conducted semi-structured phone interviews in four healthcare systems in the USA that had adopted an integrated EHR within the previous five years.

**Participants:**

Forty-two staff members of four geographically and organizationally diverse healthcare systems, including 22 individuals with formal informatics roles.

**Approach:**

Transcribed interviews were coded and analyzed using qualitative content analysis methods.

**Key Results:**

Across organizations, participants described a loss of autonomy by the EHR workforce at the individual and institutional level following the adoption of an integrated EHR. We also identified references to transformations in four key professional functions of the EHR workforce: communication, governance, optimization, and education.

**Conclusions:**

Transitions to integrated EHR systems can have important implications for the autonomy and professional functions of the EHR workforce. These findings may help institutions embarking on similar transitions better anticipate and prepare for these changes through such practices as revising job descriptions, strengthening EHR governance structures, and reinforcing pathways to engage frontline clinicians in supporting the EHR. Findings may also help institutions structure vendor contracts in a way that anticipates and mitigates loss of autonomy.

**Supplementary Information:**

The online version contains supplementary material available at 10.1007/s11606-023-08281-6.

## Introduction

Healthcare institutions in the USA are increasingly replacing their homegrown and their multi-vendor “best of breed” EHR systems with commercial integrated EHR systems in which software from a single vendor spans specialties and care settings.^1,2^ For institutions with best of breed systems, in which several EHR products from different vendors are assembled together, the unified interface of integrated EHRs may offer smoother information flow and work coordination across settings, and may even mitigate certain safety risks associated with best of breed systems.^3^ For institutions with a homegrown system, an integrated system can provide economies of scale such that the tedious technical work of sustaining a reliable software platform can be outsourced to a third party. Federal incentives to implement and achieve “meaningful use” of EHR systems may also have accelerated the move to integrated EHR systems, with vendors developing expertise in regulatory requirements and touting their ability to help their clients comply with complex and frequently changing regulations.^4^

It is already clear that EHR transitions can be profoundly disruptive, with vast ramifications for organizations’ workflows, productivity, and employee morale and well-being.^5–11^ But a dimension of EHR transitions that has not yet been well-documented is their impact on the EHR workforce within each healthcare system — namely, informatics professionals and IT staff, as well as the users and administrators engaged in supporting an EHR system and its use.^12^ Even though these individuals have been identified repeatedly as central to the success of EHR systems,^13–16^ it remains unclear how this part of the workforce is affected by EHR transitions.

In organizations with a single legacy system or a tailored combination of best-of-breed EHR products, the internal EHR workforce has typically had a significant degree of autonomy in determining what kind of documentation to promote^17,18^ and what kind of care to encourage via the EHR — for example, better targeting the use of prostate cancer screening,^19^ or encouraging family planning services when prescribing potentially teratogenic medications to women of reproductive age.^20^ However, when organizations replace their existing EHR(s) with integrated, single-vendor systems, the control that clinical and informatics leaders once had can quickly shift to their vendor. Additionally, the roles that other institutional stakeholders play in supporting the EHR may change dramatically. Though these changes have often been profound, they have been remarkably understudied. The field has begun to appreciate that the large-scale adoption of integrated EHRs can change organizational decision-making in substantial ways,^21,22^ but the nature of those changes has not been documented, and their implications for the EHR workforce within each healthcare system have not been explored. In this qualitative multisite study, we seek to address these gaps by (a) exploring how transitions to integrated EHR systems can influence institutional autonomy alongside the autonomy of the EHR workforce, and (b) describing associated transformations in professional functions of the EHR workforce. Better understanding these changes may help healthcare institutions prepare for and mitigate important challenges in EHR transitions.

## Materials and Methods

We conducted a qualitative research study in four geographically and organizationally diverse healthcare institutions that switched from homegrown or best-of-breed systems to an integrated EHR system in the prior three years. As Epic and Cerner are the current largest vendors of integrated EHRs and together account for the majority of the integrated EHR market, we included two institutions that switched to Epic, and two that switched to Cerner.

In order to support transferability of our findings, we sought to include participants from diverse institution types and sizes, with diverse roles (e.g., including leaders with formal informatics roles as well as end-users without such roles), though our analysis does not include comparisons across these sites or roles. Participants were identified via institutional websites, professional societies, and via snowball sampling. Participants were recruited by email, with $100 gift cards offered as an incentive to participate. The study was approved by the VA Bedford Healthcare System Institutional Review Board.

Semi-structured phone interviews were conducted by two trained qualitative researchers (EA, JB) between September 2019 and July 2020. The interview guide (see Appendix 1) was designed to facilitate a wide-ranging, open-ended exploration of participant experiences with the EHR transition process, including the preparation and planning for the transition, EHR customization and modification, employee well-being and proficiency during and after the transition, and perceived organizational impacts after the transition. Context-specific probes were iteratively added to the guide as the study progressed. Interviews were audio-recorded and professionally transcribed.

We used a combination of inductive and deductive qualitative content analysis methods to analyze interview transcripts.^23,24^ An initial code book included a priori concepts from literature on EHR transitions^6^ and emergent concepts identified in transcripts and interview notes. A subset of three transcripts was coded by the entire team to align coding approaches and refine the codebook. Subsequently, each transcript was coded by one investigator, and that coding was reviewed by another investigator with areas of disagreement resolved via team discussion.

During analysis, the team identified professional/institutional autonomy and transformations of the EHR workforce as important emergent phenomena that merited focused exploration. The first author then reviewed passages related to these topics and generated an initial set of themes, which were subsequently developed and refined through iterative team discussions. A more detailed account of our methods, organized according to domains from the Consolidated Criteria for Reporting Qualitative Research (COREQ) checklist^25^, is available in appendix 2.

## Results

We interviewed 42 participants (Table 1) across four institutions (Table 2). Twenty-two participants held formal roles as informatics leaders or clinical champions (e.g., superuser, EHR project champion, chief medical information officer), and the remaining 20 were end users of the EHR system without formal roles in the EHR implementation. Average interview duration was 50 min (range: 29–86 min).

**Table 1:** Site Characteristics

	Site A	Site B	Site C	Site D
Institution type	Multi-region integrated delivery system	Community hospital system	Academic medical center	Multi-region integrated delivery system
Approximate number of employees	60,000	3000	7000	60,000
New EHR vendor	Cerner	Cerner	Epic	Epic
Prior EHR type	Homegrown	Best-of-breed*	Best-of-breed*	Homegrown

^*^Best-of-breed approach combines numerous different EHRs systems for different services and functions

**Table 2:** Participant Characteristics (*n* = 42)

Characteristic	*n*
Site	
A	13
B	9
C	12
D	8
Informatics role*	
Leader or clinical champion	22
End user	20
Provider	
Yes	37
No	5
Organizational tenure	
< 2 years	0
2–5 years	11
5–10 years	4
More than 10 years	20
Not asked/declined to respond	7

^*^Formal informatics roles are often held in addition to clinical roles providing direct patient care

Our analysis generated two themes: first, a *loss of autonomy* (theme 1), with participants identifying multiple ways in which the transition to a single-vendor EHR system had impacted autonomy at the institutional as well as individual level. Second, the transitions were seen as stimulating *transformed EHR workforce functions* (theme 2), for example relating to education and governance at their institutions.

**Fig. 1:**
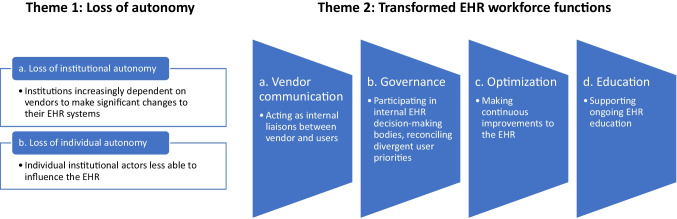
Visual representation of themes.

These themes are summarized in Fig. 1 and described below.

### Theme 1. Loss of Autonomy

Participants asserted that their *transitions to a single-vendor EHR was accompanied by losses of autonomy* both at the institutional and individual level, as described below.

#### Institutional Autonomy

Participants felt that in the wake of the EHR transition their institutions had lost direct control of their EHR systems, becoming excessively reliant on the EHR vendor. This loss of institutional autonomy was viewed as problematic on two levels. First, it was seen as an impediment to innovation, as the health systems were perceived to have less control over testing and incorporating novel features into the EHR:

**Now we’re dependent on the system**, and really that part of **not being able to make changes**, both for safety and quality, significantly makes our life very, very difficult here. (End user, B02)

The new arrangement was also perceived as less conducive to tailoring the EHR to emergent organizational needs, e.g., addressing safety concerns and quality gaps:

There were a lot of things that we wanted to do where it came down to ‘somebody at [the vendor] has to do that.’ And that was frustrating, especially coming from our perspective where **we had always had, we had local control of changes to the system**, and we could make those changes quickly and enhance the system. And **having that dependency on [vendor] employees was frustrating.** (Leader, A03)

#### Individual Autonomy

A loss of individual autonomy was also described, for leaders and front-line clinicians alike. Leaders who previously had broad authority over the EHR were held accountable for its performance, and when that authority shifted toward the software vendor, the accountability did not always shift with it:

**It’s just a very difficult job. It don’t matter if [the vendor] is unresponsive**... to the request of the institution, right? I mean, if you say, ‘hey, we need to change this,’ and they tell you, ‘well, we cannot change this for the next three months,’ well, it's a big problem … **We created a position for a chief information officer** … And he was the one in charge... with the [vendor] team, to kind of draft that implementation, right? But, unfortunately, it went so poorly, and everybody was so frustrated that **the guy immediately packed and left.** (End user, B02)

Individual clinicians also noted a loss of autonomy, albeit less directly. That is, front line clinicians noted the frustration of a facing a less-responsive party when requesting fixes or changes that they viewed as necessary. One participant explained:

We have two players, the hospital and [the vendor]... **they just point fingers to each other**... **and the clinicians and the patients are in the middle**, right? The clinicians and patients require solutions for the problem, and the solutions are not there... we have physicians that cannot put orders, cannot do notes, cannot do templates... So, bottom line, **things don’t get resolved**. (Leader, C02)

Another participant contextualized these challenges within the broader phenomenon of reduced professional autonomy in medicine overall:

As we’ve been transitioning to this more corporate model of medicine, **all of us have become more of cogs in the machine and frankly, we have to**, because the nature of medicine has changed from what it was 40 or 50 years ago. But at the same time, **one of the things that was always rewarding about medicine was a lot of autonomy, and a lot of that has gone away**. (Leader, D03)

### Theme 2. Transformed EHR Workforce Functions

While participants described a general loss of autonomy for making changes to the EHR, they also described how several *EHR-related professional functions were transformed* in more subtle ways: not necessarily losing or gaining power, but taking on a new focus in the context of a consolidated EHR. These professional functions included vendor communication, EHR governance, EHR optimization, and EHR education.

#### Vendor Communication

Participants described the emergence of an important novel role: liaison between the EHR vendor and front-line health care teams. Although vendors often have support channels (e.g., help desk portals) that can be accessed directly by clinician end users, these resources are frequently not available when needed and are staffed by personnel who may lack knowledge about issues specific to a local configuration or other important institutional context. To overcome these limitations, local liaisons were identified:

Instead of like everyone reporting all the same issues to like the [vendor] support team, we always went to our super user. And then **they would just be the go-to person to report to the [vendor] team** and find out the kinks of the system and work it out. (End user, C12)

Typically, these local liaisons were healthcare professionals embedded in clinical settings who were given protected time and formal responsibility for assisting their colleagues with the EHR and helping them navigate support resources. One participant explained how these liaisons could lower the barriers to obtaining support:

People just stop calling the help desk and placing tickets… So the whole ticket fatigue and how to report issues and get help when you need it that's not going to be a waste of your time [is helped by] **that one person you can call that can help you, and they'll put the ticket in for you**. That's been a big help. (Leader, A04)

#### Governance

Implementing an integrated EHR can change the nature of decision-making about the EHR’s clinical content. With less ability to make changes to the system directly, the opportunities to make changes (via the vendor) can become more scarce, and accordingly, participants described enhanced processes for prioritizing desired change requests. Participants also indicated a greater need for deliberative processes that engage users from all clinical specialties, clinical roles, and physical locations. One participant explained, “you’re going to be doing months and months of testing with all areas, and [you need to] ensure all areas are involved.” (Leader, C05). Another participant described a successful approach to considering user requests:

[Health system leadership] quickly developed kind of **working groups to help prioritize requests and understand where those requests were coming from** … they invited end users to participate in that, in those committees, so they could kind of review requests, and I think that was done really, really well. (End user, C10)

#### Optimization

The same interdependence that necessitates enhanced governance also demanded additional efforts to continuously test and improve the system. One participant described how after the move to a single-vendor integrated system, ongoing testing became a more urgent priority in order to ensure that all aspects of the system are working together harmoniously:

**There’s a mentality that goes into these systems that somehow you turn them on, you go through the training, you get through that phase and then everything’s going to be fine** at the other end of it. And that’s true when we had these little individual pieces, but with the way these are and the way they continue to evolve, [testing is] **definitely an ongoing process.** (Leader, D03)

Participants also explained that this testing required staff with advanced skills in extracting and analyzing data from the consolidated EHR:

When you have a problem the first thing you need is, I need to understand the problem, which means I need data about the problem, which means you need a business intelligence analyst to help you. And **unless you kind of have a lot of people around who kind of know how to get data out of [the integrated EHR] … you’re really going to struggle**. (Leader, C04)

#### Education

Particularly in the context of continuous, long-term optimization of the EHR system, participants underscored the need for ongoing user education to complement vendor-supplied training. They outlined several different important educational functions related to the EHR, including developing locally relevant educational materials, conducting EHR training focused on advanced skills, and one-on-one coaching of new hires. One participant described their approach to ongoing education about system updates and new additions:

Each department has their own meeting every other month. So at that time, **we provide education for them**, whether it’s a new protocol that’s coming up, a new power plan that’s been built in the system, **changes that are coming in the system.** (Leader, B03)

Another participant described their coaching-based strategy for training new hires:

We have kind of a pathway for people that enter the system … So this person basically **shadows the physicians all the time until they get proficient** with the system. So that's really, I think for onboarding, I think what we have now works well. (End user, B02).

## Discussion

Healthcare has been waiting for decades to achieve and reap the benefits of an integrated and interoperable network of digitized records, and many health systems and professionals have looked to the vendors of large integrated EHR systems to help realize that vision by bringing order to a messy patchwork of niche software and homegrown systems.^26,27^ However, EHR transitions are always fraught with challenges. In this analysis, we have drawn attention to a major yet underappreciated potential consequence of the adoption of large, integrated EHR systems, with a particular emphasis on their implications for the EHR workforce. Consistent with previously raised concerns about changes to professional autonomy that can accompany health IT implementations,^1,16,28–30^ we found evidence that transitions to integrated EHR systems may bring about a loss of autonomy for the institutions that adopt them and the individuals who maintain and use them.

This loss of autonomy is not without precedent. In the transition from paper records to EHRs, and especially in the adoption of clinical decision support within those EHRs, many worried that the rigidity of structured documentation and the presence of computer-based recommendations would diminish provider autonomy.^31^ In fact, the threats to autonomy explored in this study can be plausibly understood as the latest step in a long lineage of technological innovation and automation spurring far-reaching changes in professional autonomy. Long before the information age, the scientific management approach pioneered by Frederick Taylor improved productivity and reliability in manufacturing while making the multi-skilled craftsperson a less important figure, reducing the autonomy of workers, and increasing the power and centrality of managers.^31,32^ In healthcare, the professional authority and autonomy of physicians, consolidated in the early twentieth century,^33^ came to be challenged by the rise of managed care and the move towards evidence-based medicine, with administrators, particularly at larger institutions, exerting control over more and more areas of medical decision-making.^34,35^ Widespread EHR adoption both facilitated and accelerated this process, enabling extensive structured documentation, productivity and performance monitoring, and enforcing guideline compliance via EHR-embedded decision support tools.

Our work shows that a similar trajectory may be observed in the case of the EHR workforce. Whether overseeing a homegrown or a best-of-breed system, informatics and clinical leaders of healthcare institutions asserted authority over shaping the “choice architecture”^36^ that physicians and other clinical staff encountered,^37,38^ and held elite professional-managerial identities within their institutions.^39^ However, our work suggests that these institutional leaders may, too, come to cede substantial control to EHR vendors following a transition to an integrated EHR system. In this context, it is plausible that informatics leaders may seek to renegotiate and reconfigure their authority^33^ by assuming new roles related to EHR governance and support. Future research ought to shed light on how this complex reconfiguration process unfolds across health systems.

Although we focus on the potential for loss of autonomy in the transition to an integrated EHR system, it is also important to recognize the myriad factors that can threaten institutional and individual autonomy.^40^ Payers effectively constrain treatment decisions by covering certain services and not others, and by limiting coverage to a specific network of providers and facilities.^41^ Beyond coverage itself, documentation and reporting requirements from both payers and regulators limits institutional and individual autonomy in how to approach the documentation of clinical encounters, and in doing so, dictates important features of clinical workflow. Any EHR-related changes in autonomy occur against this backdrop, and the constraints imposed by payers and payment models are indeed part of the reason that changes to an EHR system can be so consequential.

Our findings have practical implications for health care institutions. An improved recognition of the potential collateral consequences of implementing integrated EHR systems may affect institutions’ process for choosing and contracting with a vendor. By anticipating the possible autonomy-reducing effects that a transition to an integrated EHR system could have, institutions may wish to structure contracts with EHR vendors in a way that sufficiently preserves autonomy in the domains that are most important to the institution.^42^ Additionally, our findings about the ways in which professional functions are transformed by transitions to integrated EHR systems may help systems better prepare — for example, by prioritizing different skillsets in recruiting health IT professionals, revising job descriptions and retraining parts of their existing EHR workforce, reimagining the roles of EHR governance structures, and reinforcing pathways to engage frontline clinicians in supporting the EHR.

Our findings, which highlight an important potential downside to single-vendor integrated EHR systems, also need to be understood in the context of the many potential advantages of such systems. Single-vendor systems have been associated with greater use of functions that enable “organizational and clinical care evaluation”^26^ and even with lower probabilities of unplanned readmission.^27^ They have also been credited with establishing “de facto interface standards” that make it easier for employees to work in and adjust to different specialty settings within an organization, and improved communication and data sharing across specialty settings within an institution.^1^ These potential advantages have made single-vendor integrated EHR systems a compelling option for many institutions, and we hope that our findings expand understanding of the less-studied but nonetheless consequential implications of these systems for the EHR workforce within each institution.

This study should be understood in the context of its limitations. Installed EHR systems are idiosyncratic and path-dependent, and the four systems in our sample may not be entirely representative of the many varieties of homegrown and best-of-breed EHR systems in place in healthcare institutions across the country. Furthermore, it is important to acknowledge that we did not interview participants at institutions that had transitioned from one single-vendor integrated system to another, nor did we explore the rarer cases of institutions switching from single-vendor systems to homegrown or best-of-breed systems. In recruiting participants, we sought to include diverse institution types and diverse employee roles, but we did not make comparisons across these many dimensions. Future studies might deepen our understanding of autonomy in EHR transitions by exploring differences by participant role, by institutional characteristics, and by EHR vendor. Future studies may also investigate institutional autonomy more directly by intentionally targeting data collection at high-level institutional decision-makers. Additionally, our findings, produced via qualitative methods, should be understood as generating, not testing, hypotheses about the implications of transitions to single-vendor integrated EHR systems. Additional research is needed to directly evaluate the impact of adopting single-vendor integrated EHR systems on institutional and individual autonomy. This research will first require the development of valid measures of autonomy that are relevant to healthcare institutions and their EHR workforces, followed by explicit measurement of institutional autonomy and individual autonomy across the many roles that comprise the EHR workforce at organizations that adopt single-vendor integrated EHR systems. Ultimately, beyond confirming or disputing the phenomenon, future research should explore ways in which healthcare institutions can retain the most important kinds of autonomy over their EHR content, and identify which functions are most important to maintain control over.

When an integrated EHR is implemented, the vendor is placed in the middle of intra-organizational negotiations and disputes about the priorities of the EHR and how those priorities are manifested in the interface. While we focus on the perspective of healthcare institutions and their providers, the incentives and perspectives of EHR vendors also merit attention. The reduced responsiveness to change requests described in our findings likely reflects vendors’ need to balance the needs of multiple diverse clients, along with their status as profit-maximizing corporations. Additional research should examine what financially sustainable steps vendors can take to adequately empower their clients while still offering a cohesive product that can be maintained and improved across institutional boundaries.^16^

## Conclusion

In this study, we unpacked the implications of EHR transitions for institutions’ EHR workforce, and the control these individuals are able to exert over their own EHR system. We found qualitative evidence of diminished institutional and individual autonomy, and we identified the nature of related transformations to the institutional EHR workforce. The increasing dominance of single-vendor integrated EHR systems may have implications on par with medicine’s transition from paper to computerized records, but those implications have only begun to be identified. Our findings highlight the need for healthcare systems and vendors to work together to develop EHR systems that achieve the key advantages of integrated EHR systems while retaining important aspects of institutional autonomy and optimizing the role of the EHR workforce within healthcare systems.

### Supplementary Information

Below is the link to the electronic supplementary material.

Supplementary file1 (PDF 77 KB)

Supplementary file2 (PDF 105 KB)

## Data Availability

The datasets generated and analyzed during this study are not publicly available because they contain information that could compromise research participant privacy. However, anonymized data extracts are available from the authors upon reasonable request.
