# Supplementary material for: From “Local Control” to “Dependency”: Transitions to Single-Vendor Integrated Electronic Health Record Systems and Their Implications for the EHR Workforce
Source: J Gen Intern Med. 2023 Oct 5;38(Suppl 4):1023–30. doi: 10.1007/s11606-023-08281-6 (PMC10593658; doi:10.1007/s11606-023-08281-6)
Supplement: Supplementary file 2 — Supplementary file2 (PDF 105 KB) [file 11606_2023_8281_MOESM2_ESM.pdf]

Appendix 2: Description of Methodology

This document provides a detailed discussion of our methodological approach in accordance with the domains of the Consolidated Criteria for Reporting Qualitative Research (COREQ).

Domain and questions	Discussion
**Domain 1: Research team and reflexivity**	
Personal characteristics	At the time of the study, all study team members were health services researchers at the Veterans Health Administration (branch of the Department of Veterans Affairs that is responsible for providing healthcare services to Veterans, their family members, and survivors). Led by a practicing physician-researcher with training in pulmonology and informatics (STR), the team was comprised of non-clinician researchers with backgrounds in medical anthropology and implementation science (EA), health services research and informatics (JB), quantitative research methods and employee wellbeing (DM), and qualitative research (ACB).<br><br>Our positionality as VA researchers directly informed the genesis of this study. In 2019, as VA's own electronic health record (EHR) transition was approaching, we decided that it was imperative to learn from the experiences of diverse non-VA sites that had undergone a similar transition in the preceding several years so as to inform VA's efforts.<br><br>All study team members were involved in developing the methodology, data analysis, and writing. Interviews were conducted by two of the study team members (EA and JB), both of whom had extensive prior experience with qualitative research in general and interviewing in particular.
Relationships with participants	Interviewers did not have a prior personal relationship with any of the interviewees, although in some cases (as explained below in the Participant Selection section), the interviewees were identified through the professional networks of other team members.<br><br>During recruitment and prior to each interview, the prospective participants were provided with the following information about the study: This is a VA study; the goal of the study is to understand the experiences of diverse health systems with EHR transitions as a way to generate knowledge about this understudied topic, as well as to inform VA's upcoming EHR modernization; the study is voluntary and confidential.<br><br>The prospective participants received the following information about the interviewers: their employment at VA, the specific VA Medical Center they were affiliated with at the time, and their role as health services researcher. No additional personal information was shared. Each interviewer also shared their professional background to the interviewees (the non-clinician status for both interviewers and lack of informatics expertise for EA) in order to orient them to potential need for extra clarification on relevant clinical and/or informatics topics during the interviews. This approach may have enriched the interviews as in some cases it may have encouraged participants to provide a more extensive amount of detail than they would have otherwise.
**Domain 2: Study design**	
Theoretical framework	We did not employ an explicit theoretical framework. Our methodological approach can best be described as qualitative content analysis with both deductive and inductive features.

Participant selection	<u>**Site selection.**</u> At the outset of the study, we determined to collect data at four organizationally and geographically diverse health systems in the United States that have undergone an EHR transition in the prior three years. Four was selected as the number that would allow us to ensure the capture of diverse organizational experiences while keeping the study feasible for our small team and short timeline. We set out to include sites that varied in the following characteristics: location, size and organizational complexity, the nature of the EHR use prior to transition (homegrown vs. commercial, single vs. multiple EHR products used), and the new EHR. Specifically, we strove to include two sites that had recently transitioned to the Cerner EHR and two that had transitioned to Epic, as these two are the dominant products on the EHR market; additionally, Cerner was already known to be the product that VA would transition to within the next year. We identified the specific sites to recruit from through our team members' prior knowledge and browsing news reports of EHR transitions.<br><br><u>**Individual participant selection**</u>**.** We used a combination of purposive and convenience sampling for including individual participants at each site. At three of the sites (A, C, and D) we made an initial contact via existing professional connections that our team members had; at the fourth site (B), we had no established connections and had to rely on a publicly available employee e-mail directory. Subsequent prospective participants were identified through suggestions from previous interviewees. We strove to recruit a diverse range of participants (frontline clinicians, leaders, individuals with an informatics role, clinicians across various primary and specialty care contexts).<br><br><u>**Saturation**</u>. We continued recruitment at each site until reaching thematic saturation (i.e., until we were unable to identify new themes pertinent to the subject of inquiry).
Recruitment	Prospective participants were sent an invitation e-mail, with up to two additional follow-ups made after the initial contact as per VA rules. IRB-approved written descriptions of the study were provided with the recruitment e-mails. At the time of the interview, the interviewer called each participant on the phone, provided another overview of the study, and ensured an opportunity to ask questions. The interviewer emphasized to each prospective participant that their participation in the study would be voluntary and that their identity and data would be kept confidential. Verbal informed consent was obtained from all participants prior to starting the interview.
Setting of data collection	Data collection was entirely virtual, with all interviews conducted over the phone. The interviewees were in the location of their choosing (workplace or home) during the interview; interviewers did not inquire or collect information about the location.
Data collection: Interview guide development	The interview guide was developed iteratively and collaboratively by the study team. No a priori theoretical framework was applied. Specific domains / question topics were determined based on team members' expertise and review of relevant literature. As many of the team members had an interest in employee wellbeing and burnout, we included questions about burnout/wellbeing in general and in the context of EHR transitions, in particular, on the interview guide.
Data collection: Interviews	**Audio/video recording**. All interviews were audio recorded and transcribed by a professional VA-approved service.

	**Field notes**. Concurrently with the interview, interviewers kept notes. After each interview, the interviewer added debrief notes to an Excel spreadsheet, containing summary of the content and emerging analytical reflections. Interviewers took special care to identify the need for any new questions or probes that might be incorporated into later interviews.<br><br>**Duration**. Interviews lasted between 29 and 86 minutes (50 minutes on average.)<br><br>**Repeat interviews**. No repeat interviews were conducted due to time limitations.
Data collection: Transcripts returned	We did not provide participants with transcripts for comment and/or correction.
**Domain 3: Analysis and findings**	
Data analysis	We used a combination of inductive and deductive qualitative content analysis methods for data analysis. We developed an initial code book based on the interview guide domains and emergent concepts derived from the review of interview notes and transcripts. As a first step, the entire team coded a subset of 3 transcripts to align coding approaches and refine the codebook. Subsequently, each transcript was coded by a single team member; the coded transcript was then reviewed by another coder; areas of disagreement were resolved via team discussion. After transcripts were coded, site-level summaries were prepared to capture each site's experiences with the EHR transition in a condensed way. During this process, the team identified professional/institutional autonomy and transformations of the EHR workforce as important emergent phenomena that merited focused exploration. The first author then reviewed the transcripts to identify any passages within and across codes that related to these topics, identifying phenomena recurring across sites, and abstracting to generate themes and subthemes. The themes were then iteratively refined through several rounds of discussion and revision in which the first author reviewed themes and subthemes, along with exemplary quotes, with the study team, and further explored the data on the basis of those discussions before revising the themes. As the final step, team members fully developed the themes by outlining their content and providing supporting quotes.
Reporting	We provided ID numbers for the interviewees after each supporting quotation. We ensured that the themes reported are consistent with the findings.
